# Improvement in catalytic activity and thermostability of a GH10 xylanase and its synergistic degradation of biomass with cellulase

**DOI:** 10.1186/s13068-019-1620-7

**Published:** 2019-12-03

**Authors:** Shuai You, Chen Xie, Rui Ma, Huo-qing Huang, Richard Ansah Herman, Xiao-yun Su, Yan Ge, Hui-yi Cai, Bin Yao, Jun Wang, Hui-ying Luo

**Affiliations:** 1 Key Laboratory for Feed Biotechnology of the Ministry of Agriculture, Feed Research Institute, Chinese Academy of Agricultural Sciences, Beijing, 100081 China; 2 School of Biotechnology, Jiangsu University of Science and Technology, Zhenjiang, 212018 People’s Republic of China; 3 Sericultural Research Institute, Chinese Academy of Agricultural Sciences, Zhenjiang, 212018 People’s Republic of China

**Keywords:** GH10 xylanase, Catalytic efficiency, Thermostability, Fragment replacement, Biomass degradation

## Abstract

**Background:**

Xylanase is one of the most extensively used biocatalysts for biomass degradation. However, its low catalytic efficiency and poor thermostability limit its applications. Therefore, improving the properties of xylanases to enable synergistic degradation of lignocellulosic biomass with cellulase is of considerable significance in the field of bioenergy.

**Results:**

Using fragment replacement, we improved the catalytic performance and thermostability of a GH10 xylanase, XylE. Of the ten hybrid enzymes obtained, seven showed xylanase activity. Substitution of fragments, M3, M6, M9, and their combinations enhanced the catalytic efficiency (by 2.4- to fourfold) as well as the specific activity (by 1.2- to 3.3-fold) of XylE. The hybrids, XylE-M3, XylE-M3/M6, XylE-M3/M9, and XylE-M3/M6/M9, showed enhanced thermostability, as observed by the increase in the *T*_50_ (3–4.7 °C) and *T*_m_ (1.1–4.7 °C), and extended *t*_1/2_ (by 1.8–2.3 h). In addition, the synergistic effect of the mutant xylanase and cellulase on the degradation of mulberry bark showed that treatment with both XylE-M3/M6 and cellulase exhibited the highest synergistic effect. In this case, the degree of synergy reached 1.3, and the reducing sugar production and dry matter reduction increased by 148% and 185%, respectively, compared to treatment with only cellulase.

**Conclusions:**

This study provides a successful strategy to improve the catalytic properties and thermostability of enzymes. We identified several xylanase candidates for applications in bioenergy and biorefinery. Synergistic degradation experiments elucidated a possible mechanism of cellulase inhibition by xylan and xylo-oligomers.
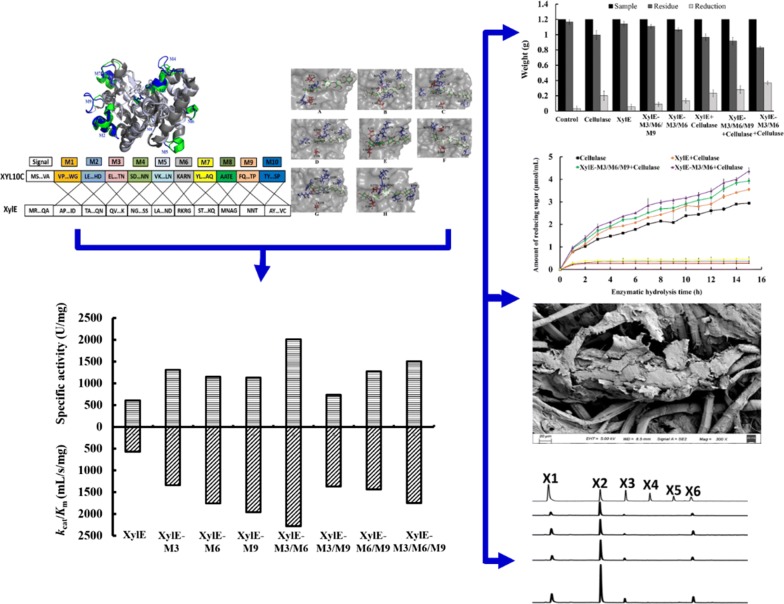

## Highlights

1. An innovative fragment-replacement-based rational design method was developed.
2. The specific activity and catalytic efficiency of the mutant xylanase reached 2010 mg/mL and 2280 mL/s/mg, respectively.
3. 4.2 µmol/mL reducing sugar was obtained from simultaneous xylanase–cellulase synergistic saccharification.
4. Comprehensive biomass utilization was realized with a tailored pretreatment and glycoside hydrolase hydrolysis strategy.

## Background

With continuous development in the global economy, people are challenged with two major social issues, environmental pollution and energy crisis. Increasing consumption of non-renewable energy has further stimulated the demand for exploring renewable energy sources [1]. Biomass is considered to be among the most promising renewable energy sources due to its energy-rich reserves and low cost of production [2]. Lignocellulosic materials, such as agricultural residues, are mainly composed of cellulose, hemicellulose, and other minor components. Its robust and complex structure makes it very difficult for enzymatic hydrolysis [3]. Therefore, the synergistic degradation of lignocellulosic biomass by cellulases and hemicellulases is an excellent strategy for efficiently converting it into resourceable sugars. Xylan is the major component of hemicellulose and represents an abundant renewable source of biomass for biorefineries [4]. Due to its heterogeneous structure, the degradation of xylan requires several types of backbone and side-chain-cleaving enzymes. Xylanases are crucial enzymes that catalyze the hydrolysis of xylan backbones, and have widespread applications in animal feed, food, bioethanol, detergent, and paper pulp industries [5, 6].

However, it is challenging to obtain a thermostable xylanase with good catalytic efficiency for industrial purposes and has been the subject of research in several disciplines [7]. Common approaches involve mining new genetic resources from extreme environments, engineering the enzymes, and optimizing their application [8, 9]. Several xylanases from *Aspergillus fumigatus* R1 [10], *Streptomyces coelicolor* A3 [11], *Aspergillus niger* [12], and *Bispora* sp. MEY-1 [13] have high catalytic activities of 18,000 to 38,000 U/mg. There have also been some successful cases of protein engineering. For example, Wang et al. [14] enhanced the catalytic efficiency of the xylanase from *Geobacillus stearothermophilus* by 3.46-fold using saturation mutagenesis and directed evolution. In addition, Wang et al. [15] enhanced the catalytic efficiency of xylanases and lichenases using oligomerization. However, there is often a trade-off between enzyme activity and stability at the level of individual mutations. In other words, enzyme rigidity is required for greater thermostability, while a flexible structure favors high catalytic activity. Therefore, mutants with increased stability often have less catalytic activity [16, 17]. Increased enzyme activity at the cost of thermostability is not biotechnologically and practically desirable. Therefore, improving the catalytic efficiency, as well as the thermostability, of an enzyme is a research focus for high-temperature industrial applications [18, 19].

Domains or peptide segments substitution has been used to improve the catalytic performance of enzymes. In contrast to site-directed mutagenesis, DNA shuffling, and random mutagenesis, which require complex calculations and laborious screening, specific fragment substitutions based on amino acid sequences and structure alignment can integrate the advantages of different enzymes. For example, Zheng et al. [20] improved the catalytic activity of cellulase BaCel5 from *Bispora* by substituting the N-terminal semi-barrel with its counterpart from TeEgl5A, while Song et al. [21] improved the substrate degradation rate of GH11 xylanase NTfus by replacing the N-terminal peptide with that of a highly active Np-Xyn. However, to date, there has been no study on the improvement in the enzymatic properties of a GH10 xylanase and its catalytic mechanism using peptide segments substitution.

In this study, fragment replacement was utilized to enhance the enzymatic properties of XylE based on sequence and structure alignments. We identified the key peptide segments influencing the thermostability and catalytic efficiency of GH10 xylanase and further elucidated their mechanism of action. In addition, we investigated the synergistic effect of cellulase and its accessory enzyme, xylanase (mutants with enhanced properties), on pretreated natural agricultural waste (mulberry bark, which contains approximately 31–33% cellulose, 17–19% hemicellulose, and 5–7% lignin). The residual dross after enzymatic hydrolysis treatment was collected to assess the effectiveness of lignocellulosic biomass hydrolysis, and then, the surface features were observed using a scanning electron microscope (SEM) to characterize the microstructure. This study thoroughly explored the mechanism by which xylanases and cellulases work together for the degradation and saccharification of lignocellulosic biomass. We have also shown that hemicellulose, especially xylan, plays a significant role in reducing the rate of enzymatic hydrolysis, which explains to some extent why the removal of hemicellulose during hydrolysis increases the saccharification efficiency of cellulase.

## Results

### Fragment identification

**Fig. 1:**
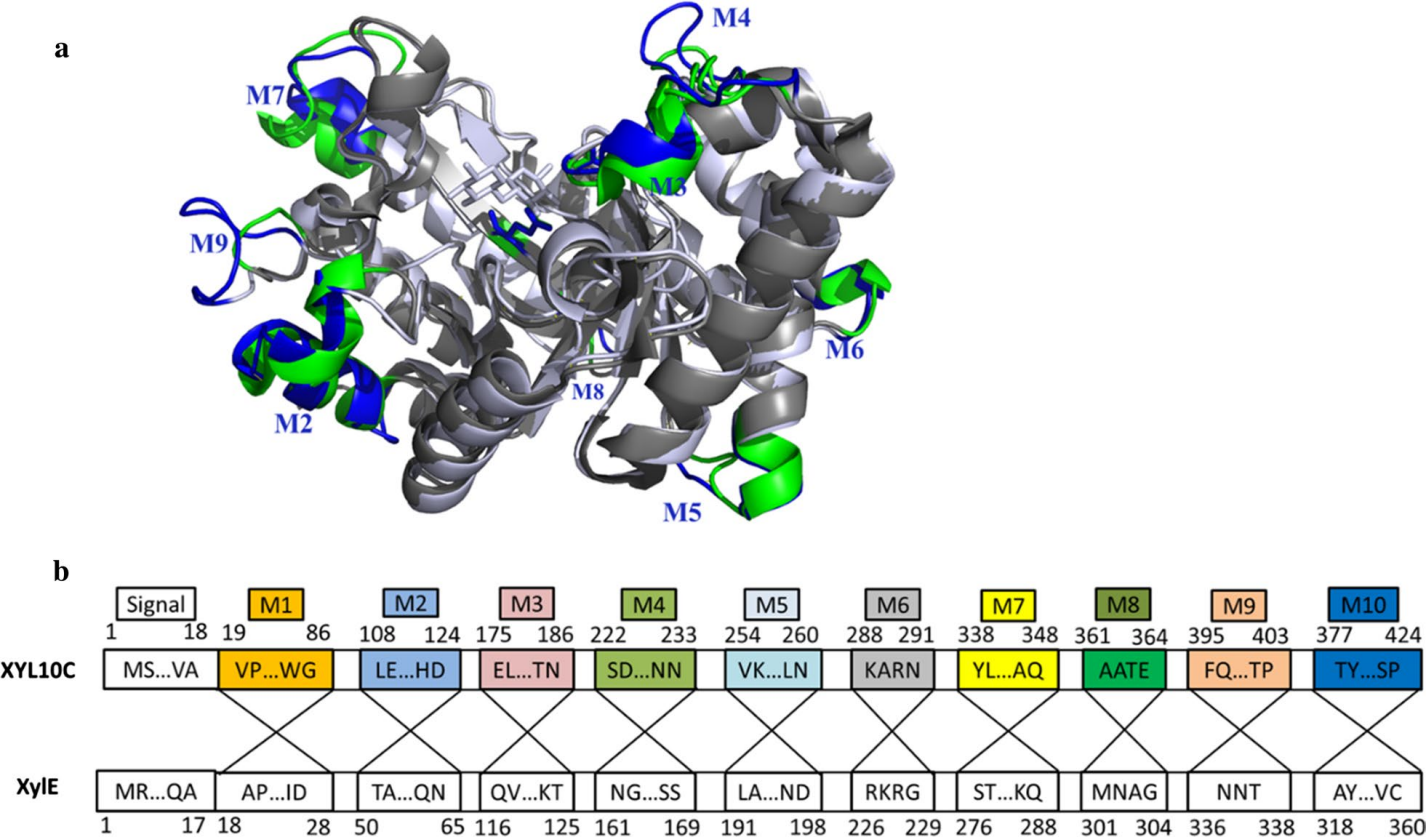
Schematic representation of the fragment replacement. **a** Structural alignment of XylE (gray) and XYL10C (silver). The peptides located on XYL10C and XylE are represented in blue and green, respectively; **b** cleavage sites on each fragment of XylE and XYL10C

The catalytic domains of XylE and thermophilic XYL10C share 53% sequence identity (Additional file 1: Figure S1), and their crystal structures showed a common (α/β)_8_-barrel fold of GH10 xylanases [22, 23]. Two standards were used to determine the demarcation point of the sequences: (1) Each sequence retains the local secondary structure and (2) except for the N- and C-terminal sequences, each sequence has a length of less than 20 amino acid residues. According to their structure alignment (Fig. 1a) and the two demarcation standards described in “Materials and methods,” we identified ten fragments. The cleavage sites on XylE for fragment substitution compared with those of XYL10C are shown in Fig. 1b.

### Production, expression, and purification of all enzymes

Wild-type XylE and its hybrid enzymes were produced in *P. pastoris* and purified to electrophoretic uniformity as described in “Materials and methods.” With beechwood xylan as the substrate, XylE and its hybrid enzyme activities were assayed at 70 °C, pH 5 for 10 min. Except for mutants XylE-M1, XylE-M2, and XylE-M10, which displayed no activity, the other mutants displayed xylanase activity. XylE-M3, XylE-M6, and XylE-M9 showed greatly increased specific activities (1130–1310 U/mg vs. 610 U/mg) compared with that of wild-type XylE. These three peptide segments were then randomly combined to generate mutants XylE-M3/M6, XylE-M3/M9, XylE-M6/M9, and XylE-M3/M6/M9. The enzymes were produced and purified as described above. SDS–PAGE analysis showed that the purified mutants XylE-M3‒M9 and the combined substitution of the key fragments had molecular masses of 43–55 kDa as compared to their theoretical values (~ 37 kDa). A single band was observed for all enzymes after treatment with Endo H, which corresponded to the theoretical molecular weight (Additional file 1: Figure S2).

### Effect of pH on the properties of XylE and its mutants

**Fig. 2:**
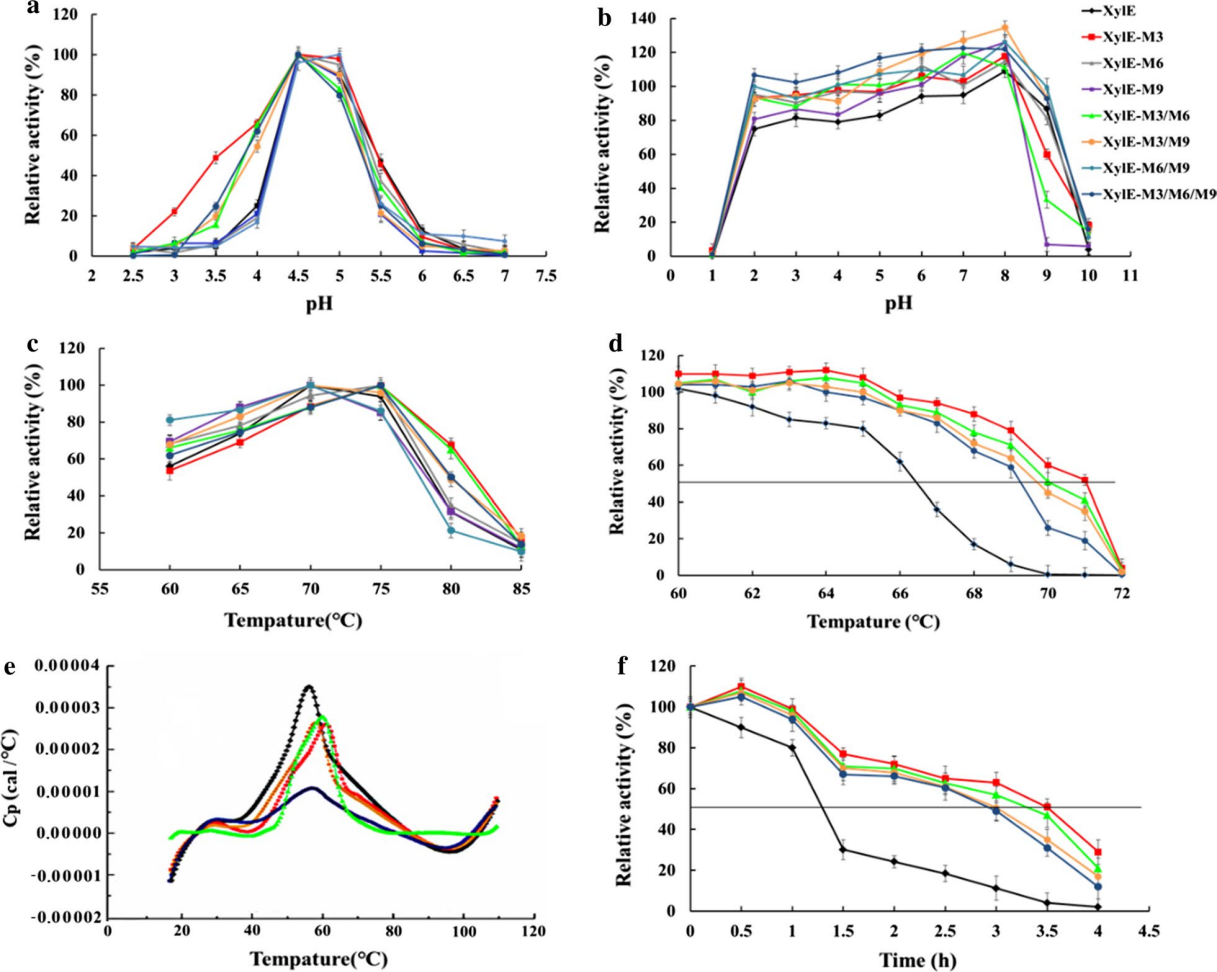
Enzymatic properties of the purified recombinant wild-type XylE and its hybrid mutants. **a** pH activity profiles tested at the optimal temperature for each enzyme (70 °C for XylE, XylE-M9, XylE-M3/M9, and XylE-M6/M9; and 75 °C for the others). **b** pH stability profiles. After incubation of the enzymes at 37 °C for 1 h in buffers ranging from pH 1 to 10, the residual activities were determined in 100 mM McIlvaine buffer at the optimal pH and optimal temperature of each enzyme. **c** Temperature activity profiles tested at the optimal pH of each enzyme (pH 4.5 for XylE and pH 5 for the others). **d** Temperature stability profiles (*T*_50_). **e** Thermograms determined by using DSC. The calorimetric recordings for XylE and its mutants were scanned at 1 °C/min in 10 mM phosphate-buffered saline (PBS) (pH 6.5) at 350 µg/mL. **f** Half-lives of wild-type XylE and its mutants at 65 °C

The effects of pH on the activity and stability of XylE and its mutants were measured using beechwood xylan as the substrate. As shown in Fig. 2a, XylE and its mutants showed maximum activity at pH 4.5 or 5, and the mutants, XylE-M3, XylE-M3/M6, and XylE-M3/M9, retained higher activities than XylE at pH 3–4 (5–66% vs. 4–27%). The results suggested that the M3 might play a role in increasing the pH range. After incubation at 37 °C for 1 h, all enzymes maintained > 70% of the highest activity at pH ranging from 2 to 8, and the mutant enzymes retained more activity than the wild type (81–135% *vs*. 75–109%) (Fig. 2b).

### Thermal properties of XylE and its mutants

As shown in Fig. 2c, the optimal temperature of XylE and its hybrid enzymes was measured over a temperature range of 60–90 °C at the respective optimal pH. Among them, the mutants, XylE-M3, XylE-M6, XylE-M3/M6, and XylE-M3/M6/M9, had similar optimal temperature of 75 °C, which was 5 °C higher than that of XylE and other mutants (70 °C). Figure 2d indicates that the *T*_50_ value of XylE was determined as 66.5 °C, while the hybrids, XylE-M3, XylE-M3/M6, XylE-M3/M9, and XylE-M3/M6/M9, had increased *T*_50_ values of 71.2, 70.4, 70, and 69.5 °C, respectively.

To investigate the thermostability of all the enzymes, their thermodynamic stabilities (*T*_m_) and half-lives (*t*_1/2_) at 65 °C were measured. The mutants, XylE-M3, XylE-M3/M6, XylE-M3/M9, and XylE-M3/M6/M9, had markedly improved thermostability. In comparison with the *T*_m_ and *t*_1/2_ values of wild-type XylE (56.1 °C and 1.2 h), mutants XylE-M3, XylE-M3/M6, XylE-M3/M9, and XylE-M3/M6/M9 had increased *T*_m_ values (by approximately 4.7, 3.6, 2, and 1.1 °C, respectively; Fig. 2e) and apparently longer half-lives (1.8–2.3 h; Fig. 2f). This result indicates that replacement of M3, alone or in combination, improves not only the pH properties, but also the thermal properties of XylE.

### Specific activity and kinetics of XylE and its hybrid mutants

The specific activity of all the enzymes was measured at a temperature of 70 °C and pH 5. Among the seven active single-fragment-substituted mutants, XylE-M3, XylE-M6, and XylE-M9 exhibited significantly higher specific activities compared with that of XylE (1130‒1310 U/mg vs. 610 U/mg). Using combined fragment replacement, the specific activity was further improved to 2010 U/mg (XylE-M3/M6). These results indicated that certain fragments might interact to improve enzyme catalysis.

The kinetic parameters of all enzymes were also measured with beechwood xylan as a substrate (pH 5, 70 °C and 5 min). All the graphs were plotted based on the Lineweaver–Burk regression and the equation that was used to calculate the *K*_m_ and *V*_max_ for each enzyme construct is shown in supplement material (Additional file 1: Figure S3). Table 1 shows that the *K*_m_ values of all the mutants were lower than that of the wild type (56–94%), indicating their increased substrate affinity. Moreover, the mutants, XylE-M3, XylE-M6, XylE-M9, XylE-M3/M6, XylE-M3/M9, XylE-M6/M9, and XylE-M3/M6/M9, showed increased catalytic efficiencies (*k*_cat_/*K*_m_), ranging from 2.4- to fourfold, compared with those of XylE. When these three peptide segments were reversibly replaced in XYL10C, no activity was detected for the mutants XYL10C-M_3_ and XYL10C-M_9_. The catalytic efficiency and specific activity of the mutant XYL10C-M_6_ were only 19% and 57% of the wild type, respectively (Table 1). The results indicated that these three peptide segments (M3, M6, and M9) have significant influence on the catalytic efficiency of the GH10 xylanases and can increase the catalytic efficiency and specific activity of the enzyme. In addition, combination of multiple fragments did not show a simple additive effect.

**Table 1:** Specific activity and kinetic parameters of XylE and its hybrid enzymes

Enzymes	*K*_m_ (mg/mL)	*V*_max_ (µmol/min/mg)	*k*_cat_ (/s)	*k*_cat_/*K*_m_ (mL/s/mg)	Specific activity (U/mg)
XYL10C	0.54 ± 0.02	3600 ± 66	2400 ± 16	4400 ± 131	3200 ± 101
XylE	0.75 ± 0.04	680 ± 31	430 ± 12	570 ± 18	610 ± 18
XylE-M3	0.61 ± 0.03	1390 ± 41	860 ± 16	1340 ± 22	1310 ± 45
XylE-M6	0.42 ± 0.01	1160 ± 17	740 ± 20	1760 ± 28	1150 ± 55
XylE-M9	0.46 ± 0.02	1390 ± 38	900 ± 10	1960 ± 31	1130 ± 38
XylE-M3/M6	0.62 ± 0.02	2200 ± 28	1410 ± 22	2280 ± 29	2010 ± 51
XylE-M3/M9	0.42 ± 0.02	880 ± 21	570 ± 16	1370 ± 22	730 ± 21
XylE-M6/M9	0.57 ± 0.01	1280 ± 34	820 ± 19	1440 ± 18	1270 ± 37
XylE-M3/M6/M9	0.67 ± 0.03	1760 ± 47	1170 ± 26	1750 ± 19	1500 ± 46
XYL10C-M_3_	ND	ND	ND	ND	ND
XYL10C-M_6_	2.02 ± 0.04	2530 ± 77	1890 ± 56	940 ± 23	1830 ± 66
XYL10C-M_9_	ND	ND	ND	ND	ND

The specific activities mentioned were calculated after conducting assays with the enzymes at 70 °C using beechwood xylan as the substrate

*ND* no activity detected

### Analysis of the hydrolyzed product

High-performance liquid chromatography (HPLC) analysis indicated that XylE and its mutants, XylE-M3, XylE-M6, and XylE-M9, hydrolyzed the β-1,4-glycosidic bonds of beechwood xylan into mainly xylose and xylobiose with trace amounts of xylotriose and xylohexaose by endo-mode of action (Additional file 1: Figure S4). The order of hydrolytic activity was XylE-M3 > XylE-M6 > XylE-M9 > XylE. These results corresponded to their order of catalytic efficiency, and this indicated that the replacement of the peptide segments had no effect on the catalytic mechanism of XylE and its mutants.

### Circular dichroism (CD) measurements

**Fig. 3:**
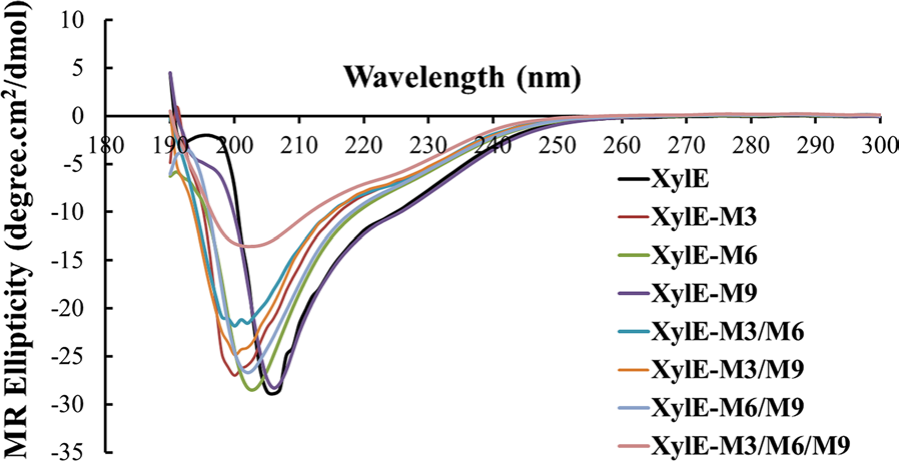
Far-UV CD spectra and RMSD values of the wild-type XylE and its hybrid mutants

CD analysis was done to check whether the enhanced catalytic efficiency of the mutant was a result of variations in the secondary structure of the protein. As depicted in Fig. 3, the far-UV CD spectrum of XylE showed a distinctive minimum and maximum at 208 and 196 nm, and these are characteristics of α-helix and β-sheet structures in aqueous solution, respectively [24]. Fragment substitution showed that the unchanged secondary structure of XylE-M9 with a far-UV CD spectrum was similar to XylE. Contrary to the other mutants, which only had one minimum peak near 198 nm, this type of structure is considered irregular [25]. These results might explain the importance of fragment substitution for improving the catalytic efficiency and thermal property of the enzymes.

### Molecular dynamic (MD) simulation

**Fig. 4:**
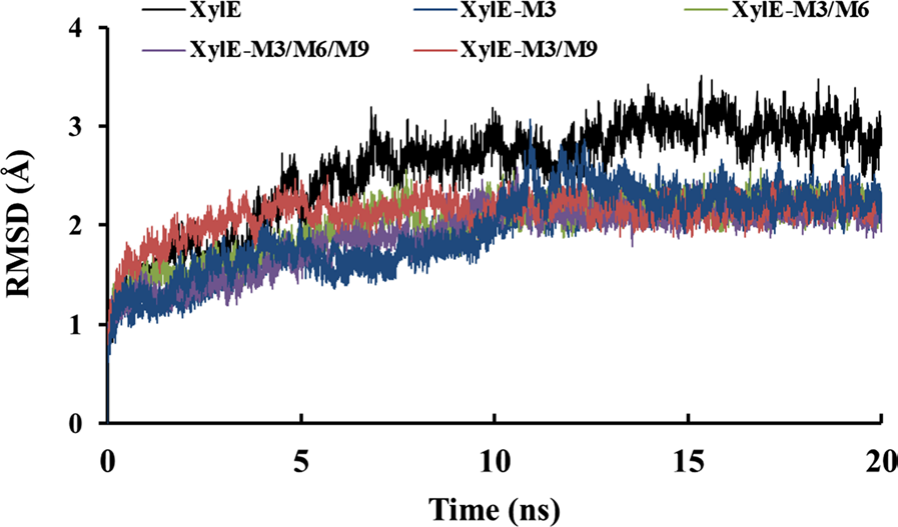
RMSD values of the wild-type XylE and its hybrid mutants. The RMSD values are shown for the XylE-M3, XylE-M3/M6, XylE-M3/M9, and XylE-M3/M6/M9 mutants calculated from the molecular dynamics (MD) simulation

To further explore the substrate binding in the catalytic sites of the enzyme, the enzyme–substrate complex was analyzed using the Discovery Studio 2017 Client. MD simulations of the wild-type XylE and hybrid mutants with the substrate molecule were used to analyze the effect of the peptide segment on ligand binding and plasticity of the protein. During the course of these MD simulations, the root mean square deviation (RMSD) of the α-carbon atoms of all the complex systems (XylE and its seven hybrid mutants with the substrate) showed stable plateaus after 15 ns (Fig. 4 and Additional file 1: Figure S5). Compared with wild-type XylE, the mutants, XylE-M3, XylE-M3/M6, XylE-M3/M9, and XylE-M3/M6/M9, showed decreased conformational flexibility, with lower RMSD values at 298 K (Fig. 4). These results are consistent with the experimental results, which showed that the thermostability of the four hybrids was markedly improved.

**Fig. 5:**
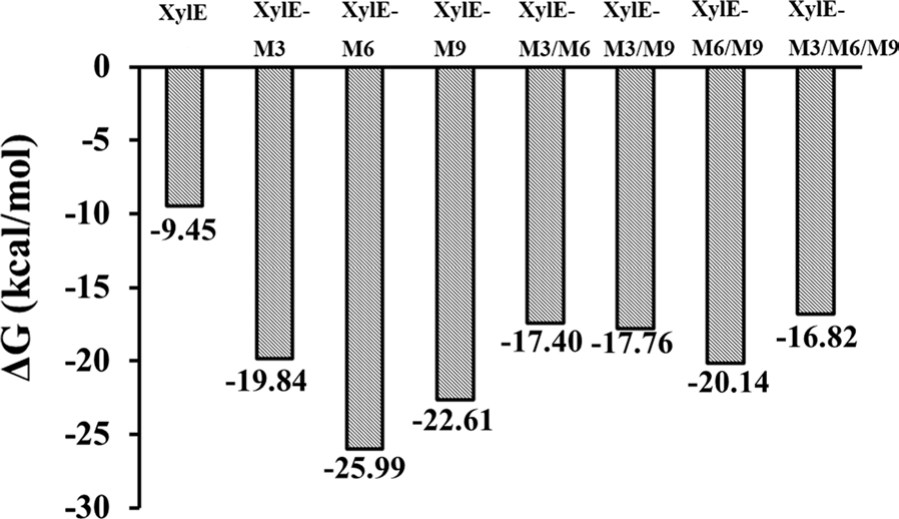
Binding free energy values of wild-type XylE and all mutants calculated using the MM/PBSA method

Binding free energy can be used to assess the sum of interactions between a protein and its ligand [26]. Based on the molecular mechanics Poisson–Boltzmann surface area (MM/PBSA) analysis, the binding free energies for the protein–ligand interactions of the mutants were much lower than that of XylE (− 16.82 to − 25.99 kcal/mol vs. − 9.45 kcal/mol; Fig. 5). The result corresponded to their *K*_m_ values. This indicated higher interactions between these hybrid enzymes and their substrates, thereby enhancing their affinity to the substrate. Further hydrogen interaction analysis indicated that XylE-M3 and XylE-M9 formed nine and eight hydrogen bonds at M3 and M9, respectively, whereas the wild-type XylE formed four and two hydrogen bonds at the corresponding fragments (Table 2). Moreover, XylE-M3 and XylE-M9 had seven and two hydrogen bonds, respectively, with higher occupancy rates than those of the wild type. Although XylE-M6 and XylE formed four hydrogen bonds at M6, three of the hydrogen bonds of XylE-M6 had higher occupancy rates than those of the XylE (Table 2). These results indicated that single substitution of M3, M6, and M9 increased local hydrogen-bonding interactions and contributed to the rigidity of the whole protein, resulting in enhanced substrate affinity.

**Table 2:** Comparison of the hydrogen bond quantity and occupancy rates of XylE and its hybrid mutants

Fragments	Residue^a^	Acceptor	Donor	Occupancy rate (%)	Acceptor	Donor	Occupancy rate (%)
M3	*116*	S_115@OG	E_116@H	11	–	–	–
	*118*	P_118@O	V_121@N	47	F_77@O	S_118@HG	45
	119	F_119@O	N_123@N	48	D_119@OD2	S_123@OG	57
	*120*	E_81@O	W_130@HE1	65	–	–	–
	121	P_118@O	F_122@H	13	S_118@O	F_122@N	28
	*122*	F_119@O	N_123@H	48	–	–	–
	*123*	P_124@O	F_126@H	15	E_130@OE2	K_124@NZ	11
	*124*	P_124@O	F_126@H	15	–	–	–
M6	*201*	V_197@O	K_201@H	91	A_237@O	R_201@NH1	83
	202	L_200@O	A_202@H	10	S_198@O	K_201@N	42
	*203*	E_153@OE2	R_203@HH12	88	E_153@OE1	R_203@HH22	75
	*204*	K_201@O	N_204@N	31	R_201@O	G_208@H	26
M9	*336*	E_346@O	F_336@H	81	E_340@O	N_336@H	64
	*337*	E_337@OD1	L_345@H	60	–	–	–
	338	P_338@O	N_342@HD22	10	N_336@OD1	F_338@HG1	37
	339	D_339@OD1	N_342@HD22	64	–	–	–
	340	G_340@O	F_343@H	24	–	–	–
	342	D_339@OD1	N_342@HD22	64	–	–	–
	343	G_340@O	F_343@H	24	–	–	–
	344	P_344@O	K_60@H21	18	–	–	–

The number of hydrogen bonds and occupancy rates are shown for the fragments M3, M6, and M9 during the last 5 ns of the trajectories

^a^Residue numbering corresponds to Fig. 2, in which the Ala18 of XylE is the first residue. The residues of wild-type XylE with lower occupancy rates are indicated in italic

### Hydrolysis of mulberry bark by cellulase and xylanase

**Fig. 6:**
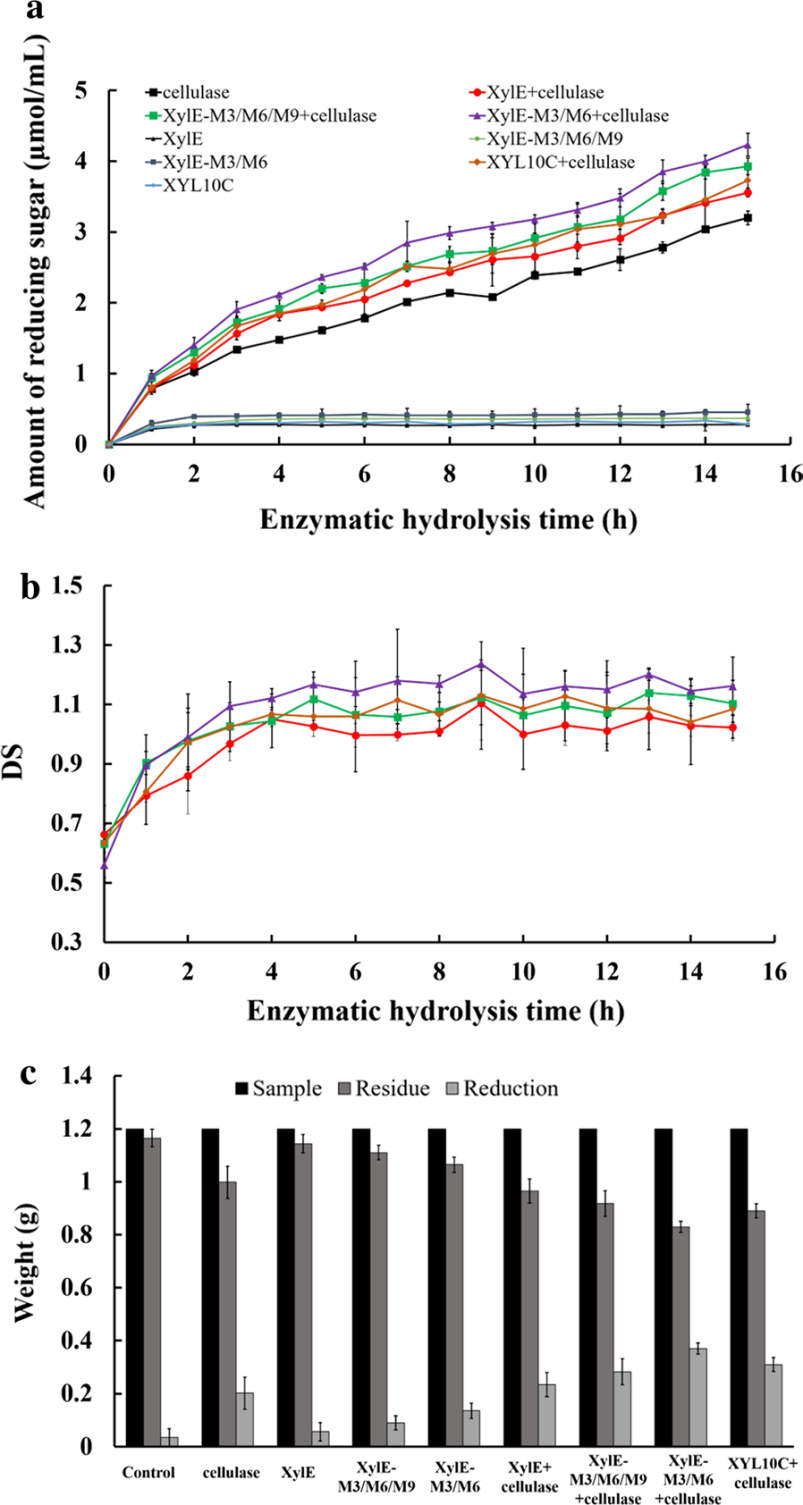
Time course hydrolysis of mulberry bark. Separate hydrolysis: 5 U each of cellulase (circle) or xylanase (triangle); simultaneous hydrolysis: 5 U each of cellulase and xylanase (inverted triangle); control: no enzyme added (square) to substrates for 15 h. The DS curve for the mulberry bark samples is shown. Enzyme loading: cellulase 5 U, xylanase 5 U. Control: no enzyme added. The changes in the dry weight of the mulberry bark during separate and simultaneous hydrolysis with cellulases and xylanase after 24 h are shown

Xylanase and cellulase were added simultaneously or individually to mulberry bark to assess the generation of reducing sugars. In this study, wild type (XylE) and two mutants (XylE-M3/M6 and XylE-M3/M6/M9) were selected to study the synergistic degradation of mulberry bark by xylanase and cellulase. As shown in Fig. 6a, the production of reducing sugar increased over time in every group as expected. The amount of reducing sugars was higher when both xylanase and cellulase were added compared with the addition of cellulase only. After 15-h incubation with both xylanase and cellulase, the reducing sugar content in the groups treated with mutants, XylE, XylE-M3/M6, and XylE-M3/M6/M9, with cellulase reached 3.6, 3.9, and 4.2 µmol/mL, respectively. Similarly, the amount of reducing sugars reached 3.2 µmol/mL in the groups where cellulase was added individually. The above results indicated that xylanase mutants promoted cellulase-catalyzed hydrolysis of mulberry bark, and the order of strength was XylE-M3/M6 > XylE-M3/M6/M9 > XylE. We also tested the reducing sugars that were formed as a result of catalysis by xylanase only. The amount of reducing sugars in mulberry bark catalyzed by XylE, XylE-M3/M6, and XylE-M3/M6/M9 was 0.28, 0.45, and 0.36 µmol/mL, respectively, after 15 h. This result indicated that ability of xylanase to hydrolyze mulberry bark was weak, and the mutant XylE-M3/M6 degraded the mulberry bark at the fastest rate, followed by mutant XylE-M3/M6/M9, which is consistent with the previously determined catalytic activity. Based on the above results, it is apparent that treatment with a combination of xylanase and cellulase increases the hydrolysis of mulberry bark more efficiently than treatment with the individual enzyme. These results also showed that the xylanase exhibited both additive and boosting effects on the activity of cellulase.

### Synergistic effect of cellulase and xylanase

In this study, we have shown the synergistic effect rather than a simple additive effect of xylanase on cellulase. The cumulative effect of xylanase on cellulase may be determined by evaluating the amount of reducing sugars produced by xylanase and cellulase treatment. In Fig. 6a, the amount of reducing sugars produced by individual treatment with cellulase was 2.9 µmol/mL, while those after individual treatment with the xylanases, XylE, XylE-M3/M6, and XylE-M3/M6/M9, were calculated as 0.28, 0.45, and 0.36 µmol/mL, respectively. The values were significantly lower than those treated with the enzyme mixture containing a combination of all the xylanases and cellulase, which were 3.6, 3.9, and 4.6 µmol/mL, respectively. Degree of synergy (DS) values were used to determine the detailed synergistic impact of xylanase on cellulose-mediated hydrolysis of biomass. According to the definition, the larger the DS, the more the amount of reducing sugars generated from addition of xylanase into cellulase. In Fig. 6b, the DS values of two mutants were all above one after 3 h, and 4 h for wild-type XylE with the highest degree of synergy of 1.3 by XylE-M3/M6, showing the synergistic effect of xylanase with cellulase. However, differences in the extent of synergy that is exhibited by the three different xylanases may well be the cause for the difference in their catalytic activities.

Furthermore, to explore the synergistic effect of xylanase on cellulase, changes in the dry weight of the substrates were determined after treating with different enzymes (Fig. 6c). Maximum loss in dry weight was shown in the mulberry bark samples treated with both mutant XylE-M3/M6 and cellulase followed by mutant XylE-M3/M6/M9 and wild-type XylE with cellulase, indicating that the addition of xylanase to cellulase can speed up the cellulose breakdown more than those treated with only cellulase or xylanase. The reduction of the substrates treated with enzyme mixtures containing XylE, XylE-M3/M6/M9, and XylE-M3/M6 in synergy with cellulase was improved by 115%, 140%, and 185%, respectively, opposed to treatment with only cellulase after 24 h. Although the production of soluble reducing sugars was not clearly detectable in the mulberry bark samples treated with only xylanase (Fig. 6a), we could clearly detect the total reduction in the dry weight (Fig. 6c). This may be due to hydrolysis of the insoluble xylans into dissolvable xylo-oligosaccharides (data not shown). We also carried out saccharification assays with XYL10C as a control as shown in Fig. 6. The experimental results showed that the synergistic effect of XYL10C with cellulase was weaker than that of XylE-M3/M6 and XylE-M3/M6/M9.

### Scanning electron microscope (SEM) analysis

**Fig. 7:**
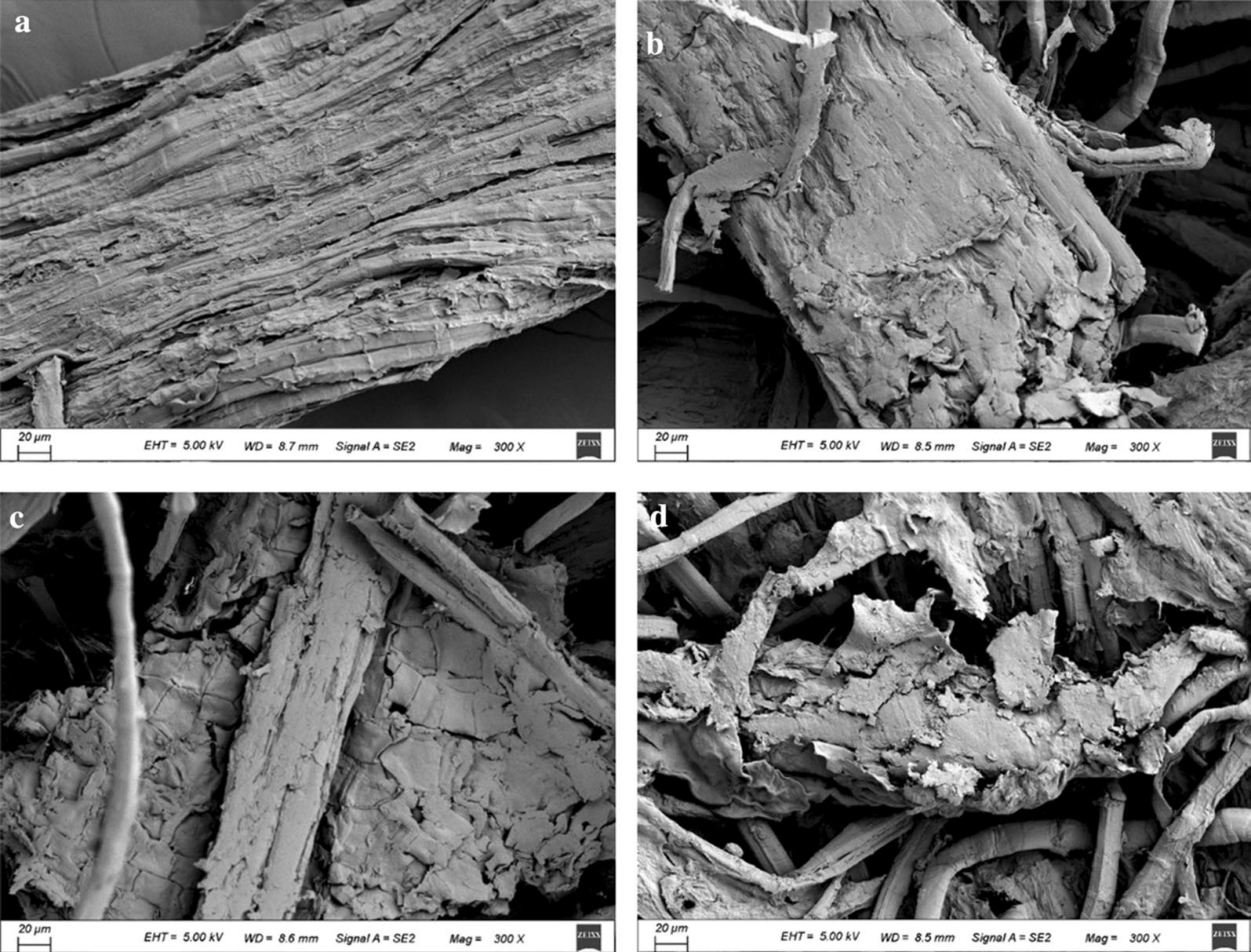
Electron microscopy images of the micro structure of the mulberry bark samples treated with different enzymes. **a** Negative control, mulberry bark treated with buffer for 24 h; The panels show the surface of the mulberry bark treated for 24 h with the following **b** XylE-M3/M6 alone; **c** cellulase alone; **d** mixture of XylE-M3/M6 and cellulase. The magnification is 300 times larger for all images

SEM was employed to monitor the changes in the surface structure of the mulberry bark samples treated with the enzymes. From Fig. 7, it is evident that the structure of the mulberry bark treated with xylanase or cellulase changed during hydrolysis. Compared with the untreated substrate (Fig. 7a), varying degrees of damage and shedding of hemicellulose were observed on the surface of the mulberry bark treated with xylanase (Fig. 7b). After 24 h of treatment with cellulase alone, the microfibers in the lignocellulose separated from each other and became loose (Fig. 7c). Contrastingly, when mulberry bark was simultaneously treated with cellulase and xylanase, the microfibers were separated from the surface hemicellulose fragments and the arrangement became more chaotic, and there was more extensive disintegration of the physical structure of lignocellulose (Fig. 7d).

## Discussion

Xylanases have attracted widespread attention due to their potential applications in various industries, ranging from food processing to kraft pulp bleaching [27]. The high catalytic efficiency and excellent stability of xylanases are prerequisites for their successful application in biotechnology. However, wild-type enzymes often do not have both favorable enzymatic properties and high catalytic efficiency, which limit their application in some industries [28]. In the studies attempting to improve the properties of these enzymes, most of the substitution of modules or regions were limited to the N terminus of the proteins [29, 30], and there are no reports showing the replacement of internal peptides in proteins, especially for the xylanases from the GH10 family. In addition, the catalytic efficiency of the modified xylanase produced using the site-directed mutagenesis method could not be greatly improved. Moreover, it also does not simultaneously take into account the thermostability and catalytic activity of the enzyme [31–33]. In the present study, we employed an internal peptide replacement method to construct hybrid enzymes. Compared to other enzyme engineering studies, this fragment substitution method is simple to operate, efficient for mutant construction, allows easy screening of target enzymes, and thus, represents an effective protein engineering approach.

XylE shares high amino acid sequence identity of 53% with the highly active, acidic, and hyperthermophilic XYL10C. However, its catalytic activity is significantly lower under acidic and thermophilic conditions, which are commonly encountered in the biorefinery industry. When we replaced each fragment of XylE with its counterpart from XYL10C, seven hybrid enzymes were produced that showed xylanase activity, while three hybrids did not. This suggested that internal peptide replacement might either create novel active enzymes or produce inactive enzymes due to improper folding, even between close counterparts. Although the peptides M3, M6, and M9 significantly contributed to the catalytic performance of XylE, substitution with their combinations had no additive effects. In general, the replacement of key peptides or residues located on the surface of a protein changes its enzymatic properties, such as the optimal conditions, catalytic efficiency, and specific activity [14, 34, 35]. Our structural analysis indicated that M3, M6, and M9 are all situated on the surface of XylE (Fig. 1a), and the replacement of these sequences might affect the overall structure of the protein. Hence, the improvement in the catalytic efficiency (*k*_cat_/*K*_m_) of XylE mutants can be attributed to the increase in the *V*_max_ and *k*_cat_ and decrease in *K*_m_. Similar observations have been reported for proteases and phytases [36, 37].

Most enzymes show altered pH activity profiles at the cost of catalytic activity. For example, Yang et al. [37] constructed six mutants of a *Bacillus circulans* xylanase and found that three of them had a different pH activity range and decreased activity (up to 70%). Given the complexity of ionic interactions, it is hard to expand the pH activity/adaptability profile of an enzyme through rational design [38]. Until now, most successful cases of shifting the functional pH range were based on random mutagenesis [39–41]. In the present study, the pH activity/stability profiles of XylE were improved by substitution with fragments from XYL10C. In particular, the replacement of M3 not only expanded the pH range in an acidic environment, but also increased the catalytic activity of the enzyme. The results indicated that the replacement of M3 might change the ionization states of the side chains of the catalytic residues [42]. Hence, our study not only improved the catalytic efficiency of XylE, but also improved its thermostability and pH stability. As a result, all the four hybrid enzymes, XylE-M3, XylE-M3/M6, XylE-M3/M9 and XylE-M3/M6/M9, showed significant improvements in their specific activity, catalytic efficiency, thermostability, and/or pH stability.

To further investigate the effect of fragment replacement on the protein structure, MD simulation analysis of wild-type XylE and its hybrid enzymes was carried out at 298 K for 20 ns. Using the RMSD value as a parameter to assess the integral structural stability of an enzyme [43], XylE, XylE-M3, XylE-M3/M6, XylE-M3/M9, and XylE-M3/M6/M9 showed marked differences compared to XylE, similar to that seen in RmXyn10A_CM, which showed enhanced thermostability and decreased RMSD values after deletion of the C-terminal flexible region [44]. This suggested that these four mutants probably have more rigid protein structures and this hypothesis was verified by the thermodynamic results. Theoretically, the MM/PBSA approach is a rigorous method for predicting the binding affinity of protein–substrate systems [26]. MM/PBSA analysis confirmed the impact of internal peptide substitution on the binding affinity of XylE and its substrates. The binding free energies of all mutants were lower than that of XylE, which indicated enhanced binding of the enzyme with the substrate favoring the catalytic process.

**Fig. 8:**
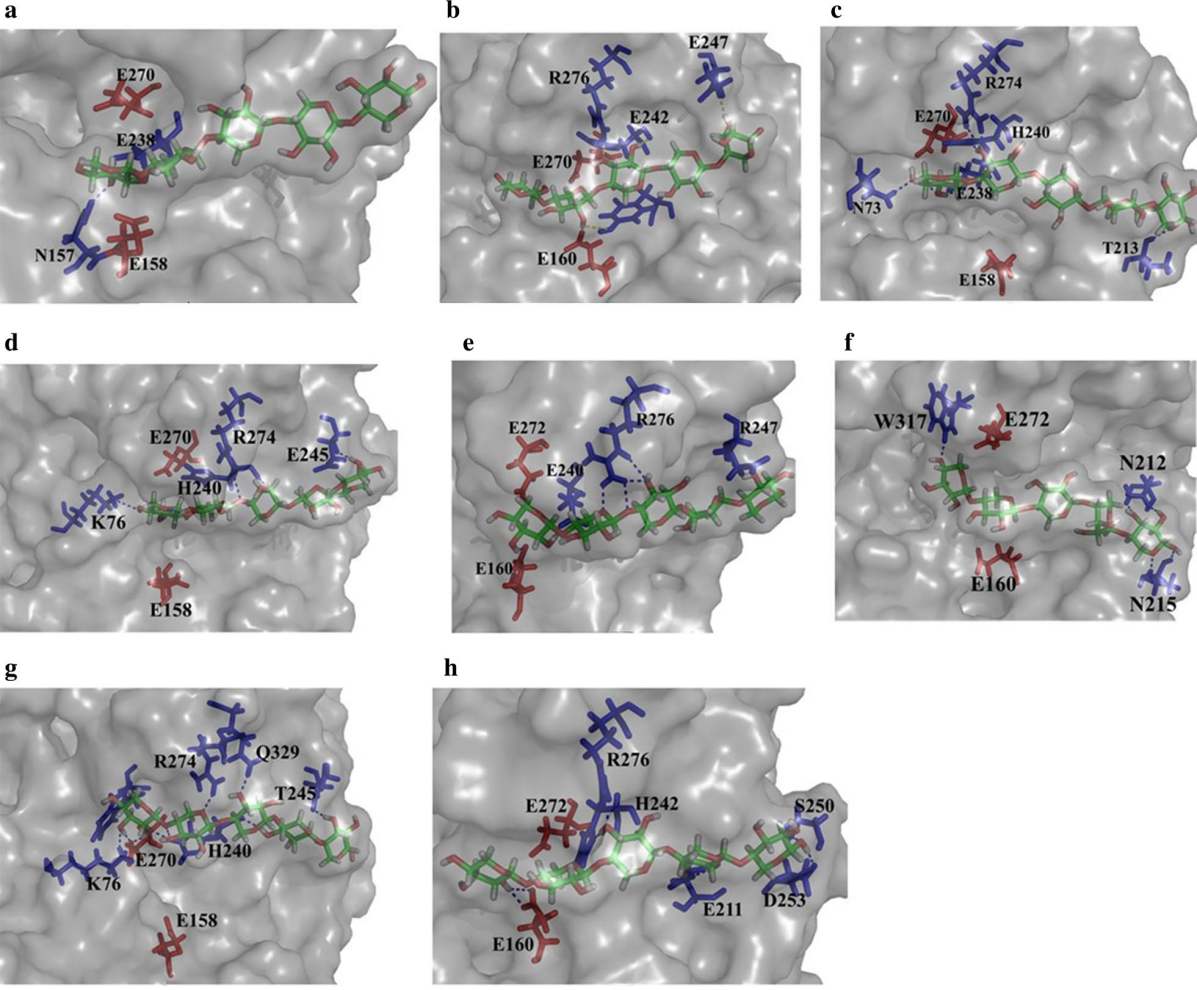
Conformational changes and interactions between wild-type XylE and its hybrid mutants after binding with xylopentaose. The figure shows the confirmations and interactions between xylopentaose and the amino acid residues in the substrate binding pocket of **a**, XylE-M3 (**b**), XylE-M6 (**c**), XylE-M9 (**d**), XylE-M3/M6 (**e**), XylE-M3/M9 (**f**), XylE-M6/M9 (**g**), and XylE-M3/M6/M9 (**h**). Catalytic residues are indicated in red; residues forming hydrogen bond interaction with the substrate are indicated in blue; xylopentaose is indicated in chromatic; and hydrogen bonds are represented by blue dashed lines

Molecular interactions, such as hydrogen bonds [45] and salt bridges [46], contribute to enzyme stability. MD analysis showed that more hydrogen bonds with higher occupancy rates were formed in M3, M6, and M9 of the mutants XylE-M3, XylE-M6, and XylE-M9 than in the corresponding fragment of wild-type XylE. These complex hydrogen-bonding networks might alter the overall protein structure and the interactions between the substrates and the enzymes. As shown in Fig. 8, the conformation of the substrate as well as the relative position of the catalytic channel and xylopentaose of the mutant enzymes changed markedly from that of the wild-type enzyme. In addition to playing a vital role in stabilizing the substrate, this type of hydrogen-bonding interaction keeps the substrate in a position that is accessible to the catalytic residues. These results further indicated that fragment replacement affects the overall structure of the enzyme and the network of the enzyme–substrate complex.

Xylanases with high catalytic activity and thermostability are required in bioenergy and biorefinery industries. Recent studies have shown that cellulases can exhibit decreased cellulose degradation rate due to adhesion to microfibrils during enzymatic hydrolysis [46]. Xylo-oligosaccharides induce cellulase inhibition, and the longer the xylo-oligosaccharides, the greater the inhibition [47]. Xylanase not only relieves the xylo-oligosaccharide-induced cellulase inhibition [48] but also helps in releasing the cellulases bound to the substrate [49]. Currently, most of the biorefinery processes use GH11 xylanase for industrial applications [50], and hence, the xylanase mutants obtained in this study can be potentially useful in biorefinery and bioenergy industries. Not only can these mutants synergistically degrade lignocellulosic substrates with cellulase but they can also increase the production as well as the total yield of reducing sugars. Xylanase acts as an auxiliary enzyme and destroys the physical structure of the surface of mulberry bark for easy cellulase–cellulose contact and also infiltrates the microfibril crevices of cellulose, thereby accelerating matrix breakdown. This synergistic effect of xylanase provides a feasible answer to reducing barriers in biomass degradation processes, which means that even if a minimal amount of enzymes is loaded onto the lignocellulosic substrate, higher amounts of reducing sugars can be produced. In this study, we have shown that xylanase XylE and its mutants XylE-M3/M6 and XylE-M3/M6/M9 might be able to remove xylan barriers that prevent the progressive movement of cellulases, including the cleavage of xylan into smaller chain xylo-oligosaccharides, which ultimately promotes degradation of lignocellulosic substrates [51–53]. Thus, the mutants obtained in this study could be potentially applied in biofuels, biomass degradation, and biorefineries.

## Conclusions

Xylanases used in biomass degradation need to possess high catalytic efficiency and excellent thermostability. Fragment substitution method was employed to produce an immobilized xylanase with improved enzymatic properties. We observed that the three internal peptides, M3, M6, and M9, alone or in combination, notably improved the catalytic performance of XylE at higher temperatures. MD simulation and free binding energy analysis revealed that fragment replacement enhanced the local hydrogen-bonding interactions and resultantly, the interaction between the substrate and the protein. We have identified the key peptides that affect the thermostability and catalytic efficiency of the GH10 xylanases. These sequences serve as useful references for studying the properties to improve the GH10 xylanase and enabling the development of an effective protein engineering strategy for potential applications in biomass degradation and bioenergy industry. The synergistic degradation experiment elucidated a possible mechanism for cellulase inhibition by xylan and xylo-oligomers, and we suggested a mechanism that explains how the addition of xylanase increases the yield of reducing sugars during the degradation of lignocellulose.

## Materials and methods

The recombinant plasmids pPIC9-XYL10C and pPIC9-XylE, which harbor the genes *xyl10C* (FJ492963) from *Bispora* sp. MEY-1 [13] and *xylE* (FJ860894) from *P. canescens* [22], were used in this work. Vectors pEASY-T3 and *Escherichia coli* Trans I-T1 were chosen for gene amplification. *Pichia pastoris* GS115 (Invitrogen, Carlsbad, CA) and pPIC9 (TransGen, Beijing, China) were used for protein expression. The cultivation and enzyme induction were conducted on the basis of the *Pichia* Expression kit instructions. The substrate beechwood xylan and standards were purchased from Sigma-Aldrich (St. Louis, MO). Restriction endonucleases, DNA polymerase, DNA purification kit, T4 DNA ligase, and endo-β-N-acetylglucosaminidase H (Endo H) were purchased from GenStar (Beijing, China). All other chemicals were of analytical grade.

### Fragment identification

The amino acid sequences and structures of XYL10C and XylE were aligned using the ClustalW software (http://www.clustal.org/) and rendered using the ESPript3.0 program (http://espript.ibcp.fr/ESPript/cgi-bin/ESPript.cgi) and PyMOL version 1.7.2.1 (DeLano Scientific), respectively. Two standards were used to determine the demarcation point of the fragments: (1) each peptide retains the local secondary structure and (2) except for the N- and C-terminal domains, each fragment has a length of less than 20 amino acid residues.

### Construction of the mutants

All the mutant genes were generated by overlap PCR using specific primers (Additional file 2: Table S1). In the first PCR step, parallel reactions were performed to amplify the target DNA fragments using the recombinant plasmids pPIC9-XYL10C and pPIC9-XylE as templates. A second PCR step was used to amplify the final DNA products using the products from the first PCR as templates and the primers XylE-F and XylE-R. The PCR products were digested using EcoRI and NotI, ligated into the pPIC9 expression vector, and sequenced. The combined substitution of key fragment was also conducted using specific primers as described above.

### Enzyme expression and purification in *P. pastoris*

Wild-type XylE and all the fragment-substituted mutants were produced in *P. pastoris*, as described previously [13]. The induced culture was collected at 12,000×*g* for 15 min and then were concentrated using a Vivaflow 200 ultrafiltration membrane with a 10-kDa molecular mass cutoff (Vivascience, Hannover, Germany), followed by desalting in 10 mM McIlvaine buffer (pH 6.5) on a fast protein liquid chromatography (FPLC) HiTrap Q Sepharose XL 5 mL column (GE Healthcare, Uppsala, Sweden). Proteins were eluted at 4 mL/min flow rate with a linear gradient of NaCl (0–1 M). The fractions displaying xylanase activity were pooled and subjected to sodium dodecyl sulfate–polyacrylamide gel electrophoresis (SDS–PAGE). All the enzymes were purified to more than 95% homogeneity. Bradford assay was used to find out the protein concentration with bovine serine albumin as a standard.

### Biochemical characterization

The xylanase activity was assayed by determining the release of reducing sugars from beechwood xylan using the dinitrosalicylic acid (DNS) method [54]. One unit of endo-xylanase activity (1 U) correlates with the release of 1 μmol of xylose equivalent for each minute under the valuation states of pH 4.5, 70 °C, and 10 min.

The determination of the pH activity of the wild-type XylE along with the hybrid mutants was conducted at the optimal temperature for each enzyme for 10 min in 100 mM McIlvaine buffer (pH 3–8). The pH stability of each enzyme was determined by measuring the residual activities under standard conditions (pH 5, 70 °C, and 10 min) after preincubation at 37 °C and pH 1–10 for 1 h. The buffers used were 50 mM glycine–HCl (pH 1–2.5), 100 mM McIlvaine buffer (pH 3–8.5), and 50 mM glycine–NaOH (pH 9–10).

The temperature for the maximal activity of the enzyme was assessed at the optimal pH (100 mM McIlvaine buffer) and at temperatures between 60 and 90 °C for 10 min. The determination of half-lives (*t*_1/2_) of the enzymes was quantified by the remaining activities under standard conditions after different periods of incubation at 70 and 75 °C in the absence of substrate, which was followed by assaying the enzyme activity under standard conditions. To determine the *T*_50_ (the temperature at which 50% of the maximal activity of an enzyme is retained), the enzymes (50 μg/mL) were incubated inside a temperature scope of 30 °C to 80 °C for 30 min in the absence of the substrate. Enzymes were put on ice immediately after heating for 10 min, and the remaining activities were then estimated under the standard conditions. All reactions were performed in triplicate.

The *K*_m_, *V*_max_, and *k*_cat_ values of the purified wild-type XylE and the hybrid mutants were carried out under standard conditions (pH 4.5, 70 °C, and 5 min) in 100 mM McIlvaine buffer containing 0.5–10 mg/mL beechwood xylan as the substrate. The records were plotted in accordance with the Lineweaver–Burk method. All reactions were conducted in triplicate.

### Differential scanning calorimetry (DSC) assay

The melting temperature (*T*_m_), which represents the thermodynamic stability of the wild-type XylE and its mutants, was determined using a MicroCal™ VP-Capillary DSC apparatus (GE Healthcare, Sweden). Each protein sample (200 μg) was dissolved in 0.5 mL of 20-mM McIlvaine buffer (pH 6.5) and loaded onto the capillary automatically. The treatment of the degassed protein and controls was done at a heating rate of 120 °C/h over the temperature range of 25–100 °C. The experiments were conducted in triplicate.

### CD analysis of the protein structure

The spectra of proteins (200 µg/mL in 10 mM sodium acetate, pH 6.5) were recorded on a Chirascan CD spectrometer (Applied Photophysics, Surrey, UK) from 190 to 250 nm, using a 1-mm cell and a bandwidth of 1 nm over three scans, at a scan rate of 120 nm/min. The results of circular dichroism analysis were analyzed using the convolutional neural network mastery (CDNN) processing software.

### Analysis of hydrolyzed product

HPLC was used to assess the capacities of wild-type XylE and its mutants to hydrolyze xylan, as described previously [55]. Purified recombinant enzymes (0.5 U) were incubated with 0.5 mg of beechwood xylan in McIlvaine buffer (pH 4.5) at 60 °C for 30 min. Samples (400 μL) were heated in a boiling water bath for 10 min to terminate the reaction and then centrifuged at 12,000×*g* at 4 °C for 10 min. Each sample (30 μL) was injected into an HPLC instrument equipped with a Carbo PacPA100 guard column (4 × 50 mm) and an analytical column (4 × 250 mm). The oligosaccharides were eluted using a mobile phase of NaOH (100 mM) at a flow rate of 1 mL/min. Xylose and xylo-oligosaccharides (X2 to X6) were utilized as standards.

### Homology modeling, molecular docking, and MD simulation

The homology modeling and structure optimization for energy minimization of all mutants were completed based on the X-ray structure of XylE (PDB: 4F8X) from *P. canescens*, performed using Discovery Studio 2017 (Accelrys Software). To analyze the enzyme and the substrate interactions, XylE and its mutants were each docked with xylopentaose conceptually using Discovery Studio 2017. The docked enzyme–substrate complexes were screened for the most reliable binding conformation, as described previously [56, 57]. The MD simulations were done utilizing the Amber 14 package at a temperature of 298 K for 20 ns. Force fields ff99SB with the TIP3P water model were used to describe the systems [58, 59]. The RMSD was determined for the protein backbone molecules utilizing minimum squares fitting. The initial 15-ns simulation was treated as the equilibration time frame, and the information of the last 5 ns was utilized for data analysis. Three-dimensional molecular visualization and figure preparation were performed using PyMOL version 1.7.2.1 and Discovery Studio 2017 Client, respectively. MM/PBSA technique was utilized to ascertain the binding free energies of the complexes of XylE or each mutant and the ligand using MD simulations [60].

### Enzymatic hydrolysis of mulberry bark

Mulberry bark was pretreated with 15% (w/w) ammonia solution at 60 °C for 24 h as reported by Li et al. [61]. The pretreated mulberry bark was dissolved in 100 mM McIlvaine buffer (pH 5) at the concentration of 4% (w/v), followed by the addition of the xylanases, XylE, XylE-M3/M6, and XylE-M3/M6/M9, and cellulase from *Aspergillus niger* [62, 63]. Then, 30 mL of the reactants was shaken at 220 rpm in a 50-mL conical flask at 50 °C for 24 h. Triplicate experiments were performed in parallel in along with a control group and the experimental groups were xylanase (5 U)-added group, cellulase (5 U)-added group, and both xylanase (5 U)-and-cellulase (5 U)-added groups. Then, 1 mL of hydrolyzed sample was collected at 1-h time intervals and was used to determine the amount of reducing sugars using DNS (3,5-dinitrosalicylic acid) method.

The analysis of the DS is determined according to the formula reported originally [64], DS = *Y*_1+2_/(*Y*_1_ + *Y*_2_). *Y*_1+2_ is the total amount of reducing sugar liberated during hydrolysis when adding xylanase and cellulase simultaneously. *Y*_1_ and *Y*_2_ represent the amount of reducing sugar liberated during hydrolysis when cellulase and xylanase are added, respectively.

### SEM analysis

After reaction completion, insoluble precipitates were collected, washed with ddH_2_O to make sure all the soluble components are removed, and then dried at 60 °C. The dried samples were sputter-coated with gold, and the surface structure was observed by a scanning electron microscope with 300× magnification.

## Supplementary information

**Additional file 1: Figure S1.** Sequence alignment of XylE and XYL10C. The fragments of M1 to M10 in XYL10C are marked in different colors. **Figure S2.** Sodium dodecyl sulfate–polyacrylamide gel electrophoresis (SDS–PAGE) analysis of the purified recombinant wild-type XylE and its mutants. Lanes: M, the standard protein molecular weight markers; A, C, E, G, I, K, N, and P: XylE, XylE-M3, XylE-M6, XylE-M9, XylE-M3/M6, XylE-M3/M9, XylE-M6/M9, and XylE-M3/M6/M9; B, D, F, H, J, L, O, and Q: the deglycosylated enzymes. **Figure S3.** Graph showing the Lineweaver and Burk regression and the equation that was used to calculate *K*_m_ and *V*_max_ for each enzymatic construction. A to J represent XylE, XylE-M3, XylE-M6, XylE-M9, XylE-M3/M6, XylE-M3/M9, XylE-M6/M9, and XylE-M3/M6/M9, respectively. **Figure S4.** High-performance liquid chromatography (HPLC) analysis of the hydrolysis products of beechwood xylan produced by the XylE (A) and its mutants. X1, xylose; X2, xylobiose; X3, xylotriose; X4, xylotetraose; X5, xylopentaose; and X6, xylohexaose. **Figure S5.** Root mean square deviation (RMSD) values of the wild-type XylE and its hybrid mutants XylE-M6, XylE-M9, and XylE-M6/M9 during the molecular dynamics (MD) simulation.

**Additional file 2: Table S1.** Primers used in this study.

## Data Availability

The dataset supporting the conclusions of this article is included within the article (and its Additional files 1 and 2).

## Abbreviations

BMGY: buffered glycerol complex medium
BMMY: buffered methanol complex medium
CD: circular dichroism
DNS: 3,5-Dinitrosalicylic acid
DS: degree of synergy
DSC: differential scanning calorimetry
Endo H: endo-β-*N*-acetylglucosaminidase H
HPLC: high-performance liquid chromatography
MD: molecular dynamic
MM/PBSA: molecular mechanics/Poisson–Boltzmann surface area
RMSD: root mean square deviation
SDS–PAGE: sodium dodecyl sulfate–polyacrylamide gel electrophoresis
SEM: scanning electron microscope
