# Supplementary material for: Improvement in catalytic activity and thermostability of a GH10 xylanase and its synergistic degradation of biomass with cellulase
Source: Biotechnol Biofuels. 2019 Dec 3;12:278. doi: 10.1186/s13068-019-1620-7 (PMC6892236; doi:10.1186/s13068-019-1620-7)
Supplement: Supplementary file 1 — Additional file 1: Figure S1. Sequence alignment of XylE and XYL10C. The fragments of M1 to M10 in XYL10C are marked in different colors. Figure S2. Sodium dodecyl sulfate–polyacrylamide gel electrophoresis (SDS–PAGE) analysis of the purified recombinant wild-type XylE and its mutants. Lanes: M, the standard protein molecular weight markers; A, C, E, G, I, K, N, and P: XylE, XylE-M3, XylE-M6, XylE-M9, XylE-M3/M6, XylE-M3/M9, XylE-M6/M9, and XylE-M3/M6/M9; B, D, F, H, J, L, O, and Q: the deglycosylated enzymes. Figure S3. Graph showing the Lineweaver and Burk regression and the equation that was used to calculate Km and Vmax for each enzymatic construction. A to J represent XylE, XylE-M3, XylE-M6, XylE-M9, XylE-M3/M6, XylE-M3/M9, XylE-M6/M9, and XylE-M3/M6/M9, respectively. Figure S4. High-performance liquid chromatography (HPLC) analysis of the hydrolysis products of beechwood xylan produced by the XylE (A) and its mutants. X1, xylose; X2, xylobiose; X3, xylotriose; X4, xylotetraose; X5, xylopentaose; and X6, xylohexaose. Figure S5. Root mean square deviation (RMSD) values of the wild-type XylE and its hybrid mutants XylE-M6, XylE-M9, and XylE-M6/M9 during the molecular dynamics (MD) simulation. [file 13068_2019_1620_MOESM1_ESM.docx]

**Additional file 1:**


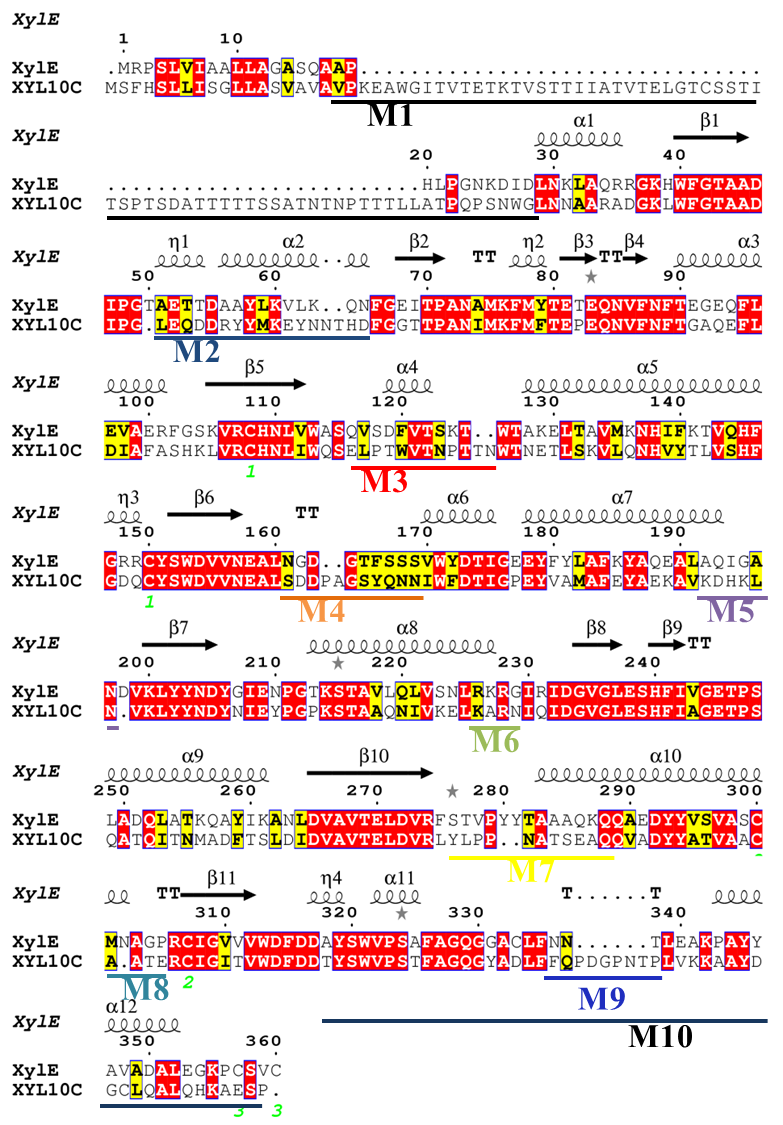


**Figure S1. Sequence alignment of XylE and XYL10C.** The fragments of M1 to M10 in XYL10C are marked in different colors.

**
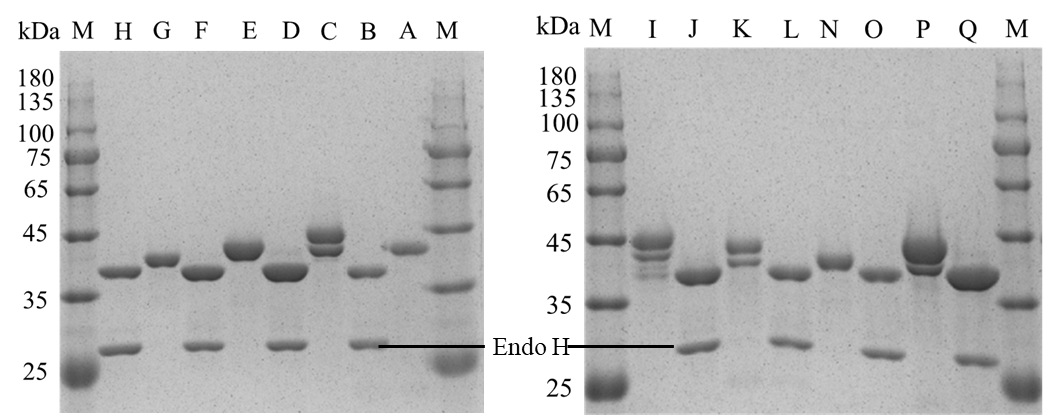
**

**Figure S2. Sodium dodecylsulfate-polyacrylamide gel electrophoresis (SDS-PAGE) analysis of the purified recombinant wild-type XylE and its mutants.** Lanes: M, the standard protein molecular weight markers; A, C, E, G, I, K, N, and P: XylE, XylE-M3, XylE-M6, XylE-M9, XylE-M3/M6, XylE-M3/M9, XylE-M6/M9, and XylE-M3/M6/M9; B, D, F, H, J, L, O and Q: the deglycosylated enzymes.


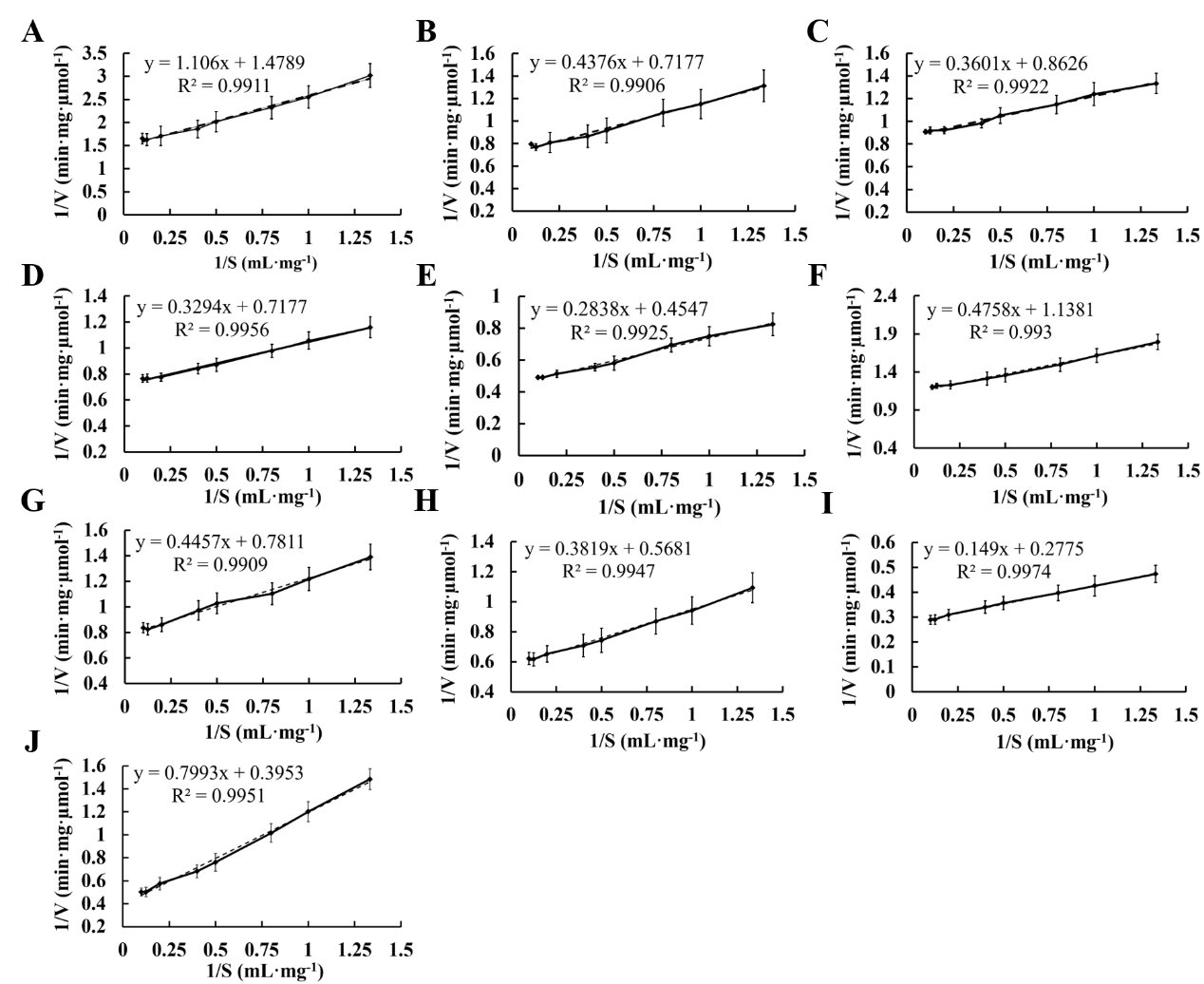


**Figure S3. Graph showing the Lineweaver and Burk regression and the equation that was used to calculate *K*_m_ and *V*_max_ for each enzymatic construction.** A to J represent XylE, XylE-M3, XylE-M6, XylE-M9, XylE-M3/M6, XylE-M3/M9, XylE-M6/M9, and XylE-M3/M6/M9, respectively.

**
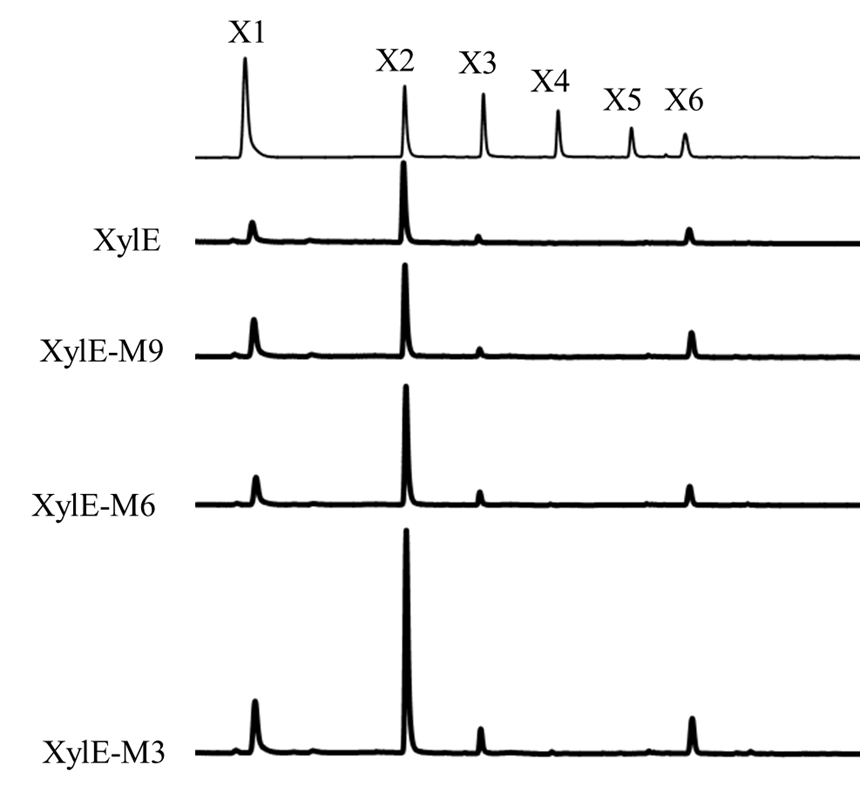
**

**Figure S4. High performance liquid chromatography (HPLC) analysis of the hydrolysis products of beechwood xylan produced by the XylE (A) and its mutants.** X1, xylose; X2, xylobiose; X3, xylotriose; X4, xylotetraose; X5, xylopentaose; and X6, xylohexaose.


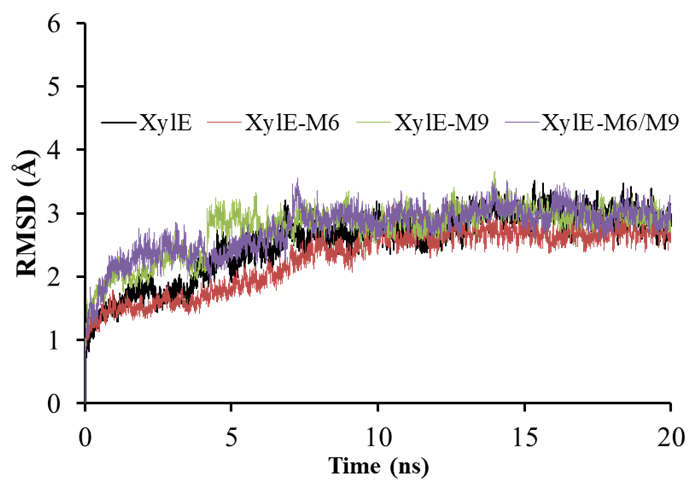


**Figure S5.** **Root mean square deviation (RMSD) values of the wild-type XylE and its hybrid mutants XylE-M6, XylE-M9, and XylE-M6/M9 during the molecular dynamics (MD) simulation.**
