# Supplementary material for: Improvement in catalytic activity and thermostability of a GH10 xylanase and its synergistic degradation of biomass with cellulase
Source: Biotechnol Biofuels. 2019 Dec 3;12:278. doi: 10.1186/s13068-019-1620-7 (PMC6892236; doi:10.1186/s13068-019-1620-7)
Supplement: Supplementary file 2 — Additional file 2: Table S1. Primers used in this study. [file 13068_2019_1620_MOESM2_ESM.docx]

**Additional file 2:**

**Table S1. Primers used in this study.**

| Primer name | Primer sequence (5′→3′) ^a^ |
| --- | --- |
| XylE-PF  XylE-PR  XylE-M1-F1  XylE-M1-R1  XylE-M2-F1  XylE-M2-R1  XylE-M2-F2  XylE-M2-R2  XylE-M3-F1  XylE-M3-R1  XylE-M3-F2  XylE-M3-R2  XylE-M4-F1  XylE-M4-R1  XylE-M4-F2  XylE-M4-R2  XylE-M5-F1  XylE-M5-R1  XylE-M6-F1  XylE-M6-R1  XylE-M7-F1  XylE-M7-R1  XylE-M7-F2  XylE-M7-R2  XylE-M8-F1  XylE-M8-R1  XylE-M9-F1  XylE-M9-R1  XylE-M10-F1  XylE-M10-R1  XylE-M10-R2 | GGGgaattcgccccacatctgcctggtaataaggacatc  GGGgcggccgcttagcaaacggaacaaggtttaccctcc  gacgaattcgtccccaaagaagcttggggaattacag  ccaggcagatgtggggcattggaaggctgcggcg  CCGCCGACATCCCAGGTTTAGAGCAGGATG  gcaggagtaatttcaccgaaatcatgcgtattg  caatacgcatgatttcggtgaaattactcctgc  catcctgctctaaacctgggatgtcggcggcagtac  cttggtttgggcttctgagcttcccacatgg  ctctttggcggtccaatttgtggtagggttag  ctaaccctaccacaaattggaccgccaaagag  catgtgggaagctcagaagcccaaaccaagttg  gtcaacgaagctttgtctgatgacccagcc  ggtgtcgtaccagatattgttttgatacgatc  gtatcaaaacaatatctggtacgacaccattgg  ggctgggtcatcagacaaagcttcgttgacaac  Gtcaaagaccataagttgaatgttaagttgtactac  attcaacttatggtctttgacggcctcttgagcgtacttg  Cttgaaagcaaggaacatcagaattgacggtgtcg  attctgatgttccttgctttcaagttggaaaccaattgc  Tgtcagattctatctgcctccaaatgctac  Tcagcctgttgggcctcgctggtagcatttg  cagcgaggcccaacaggctgaagactac  catttggaggcagatagaatctgacatcaagc  gctgcaacagaaccaagatgcatcggtgttgtcgtttg  ttctgttgcagcacaggaggcaacagagacgtagtagtc  ttccagccagacggccccaacactccccttgaggctaaa  gagtgttggggccgtctggctggaagaacaaacaggc  gatgcatcggtgttgtcactgtctgggattttg  gtagcggccgctcatggactttccgccttatgttgc  caaaatcccagacagtgacaacaccgatgcatcttg |

^a^ The restriction sites are shown underlined.
